# Effects of Bisphenol A and 4-*tert*-Octylphenol on Embryo Implantation Failure in Mouse

**DOI:** 10.3390/ijerph15081614

**Published:** 2018-07-30

**Authors:** Dinh Nam Tran, Eui-Man Jung, Changhwan Ahn, Jae-Hwan Lee, Yeong-Min Yoo, Eui-Bae Jeung

**Affiliations:** Laboratory of Veterinary Biochemistry and Molecular Biology, Veterinary Medical Center and College of Veterinary Medicine, Chungbuk National University, Cheongju 28644, Korea; mr.tran90tb@gmail.com (D.N.T.); jemman@hanmail.net (E.-M.J.); prac@naver.com (C.A.); phantom4015@nate.com (J.-H.L.); yyeongm@hanmail.net (Y.-M.Y.)

**Keywords:** implantation failure, bisphenol A, 4-*tert*-octylphenol, calcium channel

## Abstract

Miscarriage due to blastocyst implantation failure occurs in up to two-thirds of all human miscarriage cases. Calcium ion has been shown to be involved in many cellular signal transduction pathways as well as in the regulation of cell adhesion, which is necessary for the embryo implantation process. Exposure to endocrine-disrupting chemicals (EDs) during early gestation results in disruption of intrauterine implantation and uterine reception, leading to implantation failure. In this study, ovarian estrogen (E2), bisphenol A (BPA), or 4-*tert*-octylphenol (OP), with or without ICI 182,780 (ICI) were injected subcutaneously from gestation day 1 to gestation day 3 post-coitus. The expression levels of the calcium transport genes were assessed in maternal uteri and implantation sites. The number of implantation sites was significantly low in the OP group, and implantation sites were absent in the E2, ICI and EDs + ICI groups. There were different calcium transient transport channel expression levels in uterus and implantation site samples. The levels of *TRPV5* and *TRPV6* gene expression were significantly increased by EDs with/without ICI treatment in utero. Meanwhile, TRPV5 and TRPV6 gene expression were significantly lower in implantation sites samples. *NCX1* and *PMCA1* mRNA levels were significantly decreased by OP and BPA in the implantation site samples. Compared to vehicle treatment in the uterus, both the MUC1 mRNA and protein levels were markedly high in all but the BPA group. Taken together, these results suggest that both BPA and OP can impair embryo implantation through alteration of calcium transport gene expressions and by affecting uterine receptivity.

## 1. Introduction

The implantation of a blastocyst into a receptive uterus is part of a multifaceted process that includes embryo implantation, decidualization, and vascular modification [1,2], which are key events in the establishment of a successful pregnancy, and the success of each event is a prerequisite for advancement to the next stage. Blastocyst implantation occurs within a limited period when blastocyst competency is coordinated with the receptivity of the uterus. Any disturbance of this coordination can induce unsuccessful or flawed implantation. In humans, around 75% of pregnancy losses are due to implantation failure [3].

Progesterone and estrogen are the principal hormones that directly affect uterine receptivity, acting through nuclear estrogen (ERα) and progesterone receptor (PR) [1]. Both E2 and PR can regulate the production of cytokines, growth factors, homeobox transcription factors, and cyclooxygenase-derived prostaglandins. These factors are crucial during uterine preparation for implantation and post-implantation decidualization and include leukemia inhibitory factor (LIF) [4], HOXA10 (a member of developmentally regulated AbdB subclass of the homeobox gene), and the adhesion molecule mucin 1 (MUC1) [5,6,7,8,9]. Such factors are molecules that have important roles in implantation and during pregnancy [2]. Thus, the disruption of the genes associated with these factors may lead to infertility and implantation failure in various species including human. Both HOXA10 and LIF are essential for the endometrium development during implantation [10,11]. LIF and its receptor have been shown to be reduced in the endometrium of infertile women [11,12,13]. Moreover, LIF deficiency results in the loss of embryos during early pregnancy in murine [12,13,14]. A low expression of *HOXA10* mRNA results in a decreased implantation rate [15]; whereas, downregulation of *MUC1* mRNA and its protein prior to implantation allows the embryo to attach to the maternal uterus [5,16,17]. Furthermore, LIF and MUC1 were shown to enhance the calcium ion level in in vitro experiments [18,19].

Calcium ion has important roles in many aspects of a living organism. However, the role of the calcium ion in female reproductive organs is not fully described. During pregnancy, calcium ion is involved in a variety of crucial process including fertilization, decidualization, and implantation [20,21,22]. The transient receptor potential cation channel, subfamily V (TRPV) member 5, as well as TRPV6, the Na^+^/Ca^2+^ exchanger 1 (NCX1), and the plasma membrane Ca^2+^-ATPase (PMCA1) appear to have critical roles in calcium ion absorption [23]. These proteins are found in the apical membranes of intestinal and renal epithelial cells and have been proposed as mediators of calcium ion uptake during transcellular transport [24]. Recently, we reported that the calcium ion influx genes *TRPV5* and *TRPV6* are expressed in the uterine endometrial region, while the calcium ion efflux genes *NCX1* and *PMCA1* were detected in the basolateral membranes of the uterus [25]. These observations suggest that these calcium ion transport genes not only have a critical role in calcium ion transport in the duodenum and kidney but also in the uterus. In addition, TRPV6 has been reported to be a key element in controlling calcium transport between the embryo and placenta in uterus during pregnancy [26,27]. Furthermore, TRPV6 and PMCA1 are involved in specific uterine functions, including fetal implantation in human [28]. However, the roles of the calcium channel during the implantation stage have not been elucidated.

During the last few decades, there has been a growing concern about the effect of exposure to environmental endocrine-disrupting chemicals (EDs) on the reproductive system. Recent studies have shown that EDs are associated with adverse reproductive health outcomes in human, including infertility, implantation failure, and pregnancy loss [29,30]. bisphenol A (BPA; 4,4′-(propane-2,2-diyl)diphenol) and 4-*tert*-octylphenol (OP; 4-(1,1,3,3-tetramethylbutyl)phenol) are common EDs and are reported to have weak estrogenic activities. Exposure to BPA and OP during early pregnancy has resulted in implantation failure [31,32,33]. Moreover, our recent study showed that both BPA and OP could modulate the calcium channel during pregnancy [34,35]. In addition, BPA exposure had increased the expression of MUC1 [36] and decreased the expression of HOXA10, both at the mRNA and protein levels [37], during early pregnancy. Thus, we hypothesize that BPA and OP, through their estrogenic activity, can affect the calcium channel and disrupt the expression of pregnancy-related genes such as *HOXA10*, *LIF*, and *MUC1* during the implantation stage. In addition, previous studies have indicated that these genes are expressed in endometrium or are regulated for endometrial receptivity in the uterus. Regardless, successful implantation requires the synchronized development of both the embryo and the endometrium. In the present study, we investigate the effects of EDs on both the uterus and the blastocyst implantation site.

## 2. Materials and Methods

### 2.1. Chemical

BPA and 17β-estradiol were purchased from Sigma Chemical Co., (St. Louis, MO, USA), while ICI 182,780 (ICI) was obtained from Tocris Bioscience (Bristol, UK). OP was obtained from Fluka Chemie (Seoul, Korea). Stock solutions were diluted with corn oil (Sigma-Aldrich, St. Louis, MO, USA). Chicago Skye Blue 6B was bought from Sigma Chemical Co. 

### 2.2. Animals and Treatments

Female ICR mice (9-weeks-old, 25–30 g) were purchased from Samtaco (Osan, Gyeonggi, Korea). Mice were housed in polycarbonate cages under controlled environment conditions with a 12 h light/dark cycle, a constant temperature of 23 ± 1 °C, and a relative humidity of 50 ± 10%. The mice were fed a diet of AIN-76A and tap water. After a 1-week adaption period, female mice were mated with adult ICR male mice overnight, and the presence of a vaginal plug was checked the following morning. The day a vaginal plug was observed was set as gestation day (GD) 0.5.

From GD 0.5 to GD 3.5, the pregnant mice were randomly divided into seven groups (*n* = 8 mouse/group). Mice were given subcutaneous (s.c.) injection of corn oil (vehicle (VE) group), or ICI (4 mg/kg), or estradiol (E2; 40 µg/kg/day; positive control group), or BPA (100 mg/kg/day), or OP (100 mg/kg/day) dissolved in corn oil (Sigma-Aldrich). Mice in three additional groups (E2 + ICI, BPA + ICI, and OP + ICI) received s.c. injection of ICI. (4 mg/kg) 30 mins before treatment with E2, BPA, or OP. BPA-exposed mice during preimplantation in which 100 mg/kg BPA did not produce the loss of implantation sites [38,39,40]. A similar OP dose was selected to allow comparison with the BPA results. Five mice of each group were sacrificed 24 h after final treatment (GD 4.5) and uterus tissue was collected. The remaining three mice were sacrificed 3–5 min after Chicago Blue dye injection on GD 5.5. In all groups, the implantations were counted, and implantation tissue samples were collected. All animal experimental procedures were approved by Chungbuk National University Institutional Animal Care and Use Committee (IACUC) (project identification code: CBNUA-1108-17-01, approval date 1 July 2017).

### 2.3. Total RNA Extraction and Quantitative Real-Time PCR

Total RNA was extracted from uterus and implantation site samples by using Trizol reagent (Ambion, Austin, TX, USA) and the concentration of total mRNA was determined by measuring the absorbance at 260 nm. First-strand complementary DNA (cDNA) was produced from 1 µg of total mRNA by reverse transcription (RT) using Moloney murine leukemia virus reverse transcriptase (iNtRON Bio, Gyeonggi-do, Korea) and random primers (9-mers; TaKaRa Bio., Shiga, Japan). The cDNA template (1 µL) was assayed by applying SYBR-ROX (TaKaRa Bio) real-time PCR according to the manufacturer’s protocols. Primer sequences were presented in Supplementary Table. The qRT-PCR was carried out for 40 cycles using the following schedule: denaturation at 95 °C for 30 s, annealing at 58 °C for 30 s, and extension at 72 °C for 30 s. Fluorescence intensity was measured at the end of the extension phase of each cycle. The threshold value for the fluorescence intensity of all samples was set manually. During the exponential phase of PCR amplification, the reaction cycle at which PCR products exceeded the fluorescence intensity was the threshold cycle (CT). The CT value was determined automatically from the exponential phase of the delta (Δ) CT fluorescence detection graph. The expression of a target gene was quantified relative to that of the internal vehicle gene (18S ribosomal RNA) based on a comparison of CTs at a constant fluorescence intensity. Expression of 18s was not significantly altered under all experimental conditions. The amount of transcript present was inversely related to the observed CT, and the CT was expected to increase by one for every 2-fold dilution in the amount of transcript. Relative expression (R) was calculated using the equation R = 2^−(ΔCTsample − ΔCTcontrol)^. To determine a normalized arbitrary value for each gene, every data point was normalized to the control gene, as well as to the respective controls.

### 2.4. Western-Blot Analysis

Proteins were extracted from mouse uterus and implantation site samples by using Pro-prep solution (InTron, Seoul, Korea) according to the manufacturer’s protocol. A 20 µg cytosolic protein sample was separated by using 10% sodium dodecyl sulfate-polyacrylamide gel electrophoresis (SDS-PAGE) and the product transferred to a polyvinylidene fluoride (PVDF) membrane (Merck Millipore, Taunton, MA, USA). The membrane was then blocked in TBS-T containing 5% skim milk for 60 min, then incubated overnight in primary antibodies for: TRPV5 (Santa Cruz Biotechnology, Dallas, TX, USA, dilute 1:3000), TRPV6 (Alomone labs, Jerusalem, Israel, catalog: ACC-036, diluted 1:3000), PMCA1 (Swant, Marly, Switzerland, dilute 1:3000), NCX1 (Abcam, Cambridge, UK, dilute 1:3000), MUC1 (Abcam, dilute 1:3000), or β-actin (Cell Signaling Technology, Danvers, MA, USA, diluted 1:3000). After washing with TBS-T buffer, the membranes were incubated with the appropriate horseradish peroxidase-conjugated secondary antibodies (anti-rabbit, Cell Signaling Technology 1:3000; anti-mouse, Cell Signaling Technology 1:3000; anti-hamster, Jackson Human Research, West Grove, PA, USA, 1:300) for 1 h at room temperature. The membrane was then washed four times for 10 min each with TBS-T. Enhanced chemiluminescence (ECL) reagent (Santa Cruz Biotechnology) with a charge-coupled device was used to detect antibody binding. Using the Chemi Doc equipment, GenGnome 5 (Syngene, Cambridge, UK). The optical density of the target band was analyzed by using Image J software (NIH, Bethesda, MD, USA).

### 2.5. Statistical Analysis

All statistical analyses were performed by applying two-way ANOVA. Data were analyzed using GraphPad Prism software (v.4.0; GraphPad Software, La Jolla, CA, USA). The results are presented as means ± SEM. A *p* < 0.05 was considered statistically significant. All combination treatment group results were compared to the VE or ICI group and the individual treatment group results.

## 3. Results

### 3.1. Effect of EDs on Blastocyst Implantation

**Figure 1:**
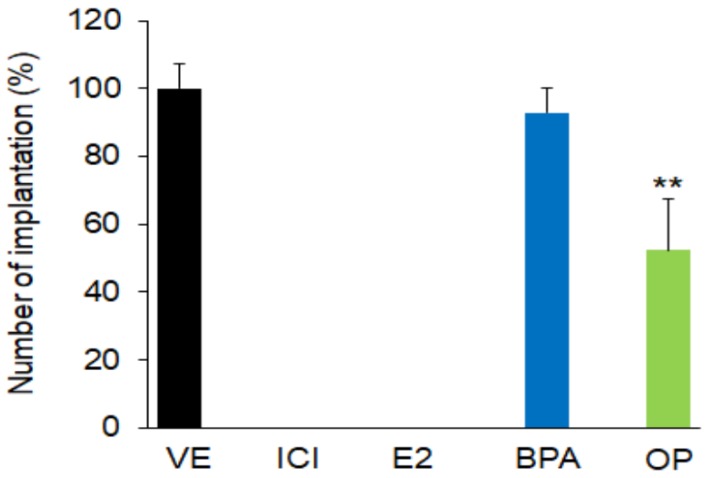
E2, BPA, and OP result in loss of implantation sites. Pregnant mice at gestation day 5.5 (GD 5.5) were sacrificed 48 h after final injection. Implantation sites in uteri were detected by application of Chicago Sky Blue 5 min before sacrifice. All implantation sites in the control group were detected as distinct blue bands. The number of implantation sites was significantly low in the OP group and there were no sites in the ICI and E2 groups. *n* = 3 mice per group. Statistical significance was determined by one-way ANOVA with the Bonferroni correction test. ** *p* < 0.01 vs. VE group. VE: vehicle; BPA: bisphenol A; OP: 4-*tert*-octylphenol; E2: 17β-Estradiol.

Initially, we examined the effect of BPA and OP on embryo implantation. Statistical analysis performed on the combined data from GD 5.5 showed that the number of implantation sites in pregnant mice treated with OP (100 mg/kg) from GD 0.5 to GD 3.5 was significantly lower than that in VE-treated mice. There was no significant difference between the numbers of implantation sites in the BPA (100 mg/kg) and VE groups (Figure 1), but the implantation sites had different appearances in the two groups. Both BPA and OP substantially reduced implantation site growth and size (Supplementary Figure S1). 

There were no implantation sites observed when mice were treated with ICI, E2, E2 + ICI, BPA + ICI, or OP + ICI. In previous study, ICI-alone exposure inhibited the process of embryo implantation in GD5 [41]. These suggest that both BPA and OP affected blastocyst implantation and the complete loss of implantation in BPA or OP with ICI exposure may due to ICI effect. 

### 3.2. Effects of BPA and OP on TRPV5, TRPV6, PMCA1, and NCX1 Expressions in Maternal Uterus and Implantation Sites

**Figure 2:**
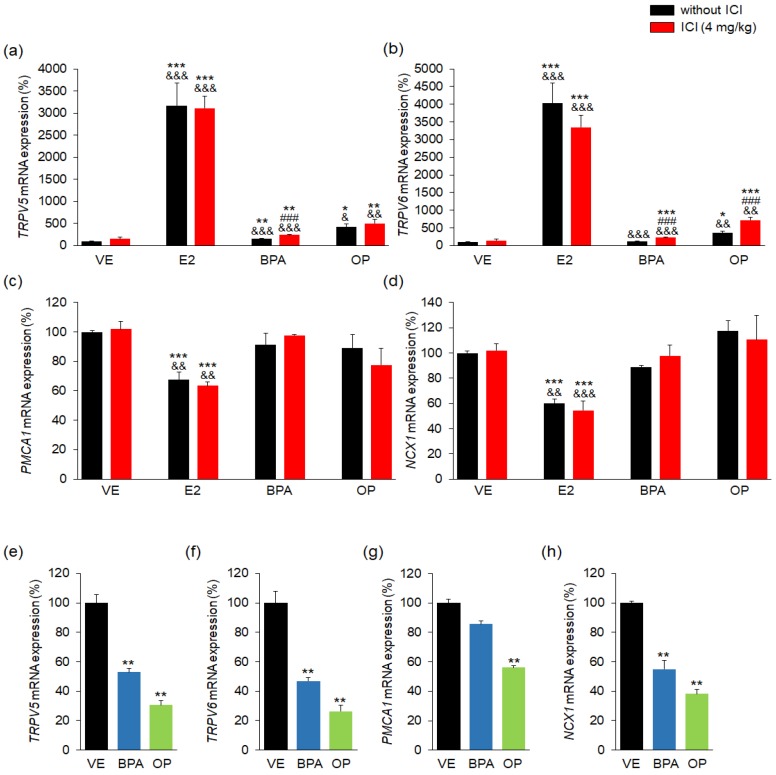
E2, BPA, and OP change the expressions of *TRPV6*, *TRPV5*, *PMCA1*, and *NCX1* in maternal uterus and implantation sites. Mice were sacrificed at GD 4.5 (24 h after final injection) to collect uterus tissues and at GD 5.5 (after 48 h after final injection) to collect implantation sites. The mRNA expressions of calcium transporter channel genes in uterus and implantation sites were assessed. The mRNA levels of *TRPV6*, *TRPV5*, *PMCA1*, and *NCX1* genes were measured by using real-time PCR and were normalized to that of 18S ribosomal RNA (RN18S). In uterus, (**a**) the expressions of *TRPV5* mRNA were significantly high in the E2, E2 + ICI, BPA + ICI, OP, and OP + ICI groups; (**b**) *TRPV6* mRNA level changes were similar to those for *TRPV5* expression. mRNA expression of *TRPV5* and *TRPV6* in uteri were higher in BPA + ICI group than in the BPA group; (**c**,**d**) mRNA level of *PMCA1* and *NCX1* were significantly decreased by E2 and E2 + ICI. In implantation sites; (**e**–**h**) the mRNA levels of *TRPV6*, *TRPV5*, *PMCA1*, and *NCX1*, respectively, were markedly low in all groups. Statistical significance was determined by two-way ANOVA. * *p* < 0.05 vs. VE, ** *p* < 0.01 vs. VE, *** *p* < 0.001 vs. VE, ^###^ *p* < 0.001 EDs + ICI vs. EDs, ^&^ *p* < 0.05 vs. ICI, ^&&^ *p* < 0.01 vs. ICI, ^&&&^ *p* < 0.001 vs. ICI. *n* = 5 mice per group for uterus, *n* = 3 mice per group for implantation sites. Treatments: E2; 40 µg/kg/day, BPA; 100 mg/kg, OP; 100 mg/kg, ICI; 4 mg/kg.

To determine the effects of BPA and OP on the regulation of calcium ion transport during implantation in the uterus and at implantation sites, TRPV5, TRPV6, PMCA1 and NCX1 expression levels were quantified by both real-time-PCR and western-blot assays. In uterus samples, there were marked increases in mRNA levels of *TRPV5* in the E2, E2 + ICI, BPA, BPA + ICI, OP, and OP + ICI groups (around 3171%, 2267%, 157%, 236%, 420% and 500%, respectively) over that in the VE group (Figure 2a). 

The *TRPV6* mRNA levels were also higher in the E2, E2 + ICI, BPA + ICI, OP and OP + ICI groups (by 4023%, 3343%, 224%, 360% and 719%, respectively) than in the VE group (Figure 2b). Furthermore, TRPV5 and TRPV6 protein levels were markedly higher in all groups than that in the VE group (Supplementary Figure S2a,b).

The expressions of *TRPV5* and *TRPV6* mRNA in uteri were higher in BPA + ICI group than in the BPA group. In addition, *TRPV6* mRNA in uteri were higher in OP + ICI group than in the OP group. The expressions of *PMCA1* and *NCX1* mRNA were significantly lower in the E2, and E2 + ICI groups (around 30% lower) than in the VE and ICI groups (Figure 2c,d). There was no difference in PMCA1 protein levels (Supplementary Figure S2c). In contrast, NCX1 protein levels were significantly high in the E2 group (Supplementary Figure S2d). 

The mRNA expression levels of *TRPV5*, *TRPV6*, *PMCA1*, and *NCX1* were markedly low in the BPA- and OP-treated implantation sites (Figure 2e–h). Moreover, the protein levels of TRPV5 and TRPV6 were significantly low in the OP group (Supplementary Figure S2e,f). Treatment with BPA also decreased the TRPV5 and TRPV6 protein levels but not significantly (Supplementary Figure S2e,f). Protein levels of PMCA1 and NCX1 were also not significantly decreased by BPA or OP treatment (Supplementary Figure S2g,h). These results suggest that BPA and OP treatments lead to abnormal expressions of calcium channel genes in both uterus and implantation sites.

### 3.3. Effect of BPA and OP on MUC1 Expression in Maternal Uterus and Implantation Sites

**Figure 3:**
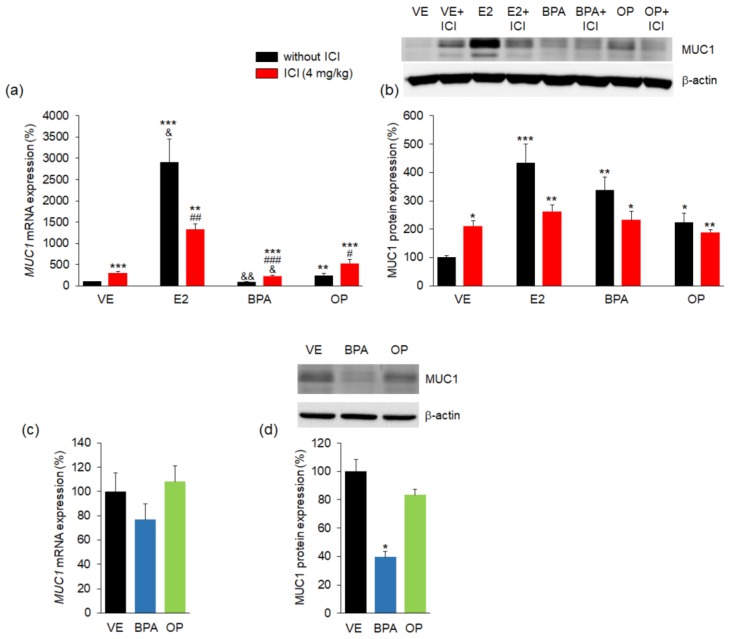
BPA, OP, and E2 increased the expression of *MUC1* in maternal uterus and implantation sites. The expression of *MUC1* was measured by real-time PCR and was normalized to that of 18S ribosomal RNA (RN18S). Expression of MUC1 protein was investigated by western blotting and was normalized by β-actin. Histograms show quantification of western blots. In uterus, (**a**) the expressions of *MUC1* mRNA in maternal uterus were markedly high in the E2, OP, and EDs + ICI groups; (**b**) Protein levels of MUC1 were significantly high in all administered groups. mRNA levels of MUC1 were significantly higher in the combination BPA + ICI and OP + ICI than that in BPA- and OP-alone; In implantation sites, (**c**) there were no differences in the expression of *MUC1* mRNA; (**d**) Protein level of MUC1 was significantly decreased by BPA treatment. Statistical significance was determined by two-way ANOVA. * *p* < 0.05 vs. VE, ** *p* < 0.01 vs. VE, *** *p* < 0.001 vs. VE, ^#^ *p* < 0.05 EDs + ICI vs. EDs, ^##^ *p* < 0.01 EDs + ICI vs. EDs, ^###^ *p* < 0.001 EDs + ICI vs. EDs, ^&^ *p* < 0.05 vs. ICI, ^&&^ *p* < 0.01 vs. ICI. *n* = 5 mice per group for uterus, *n* = 3 mice per group for implantation sites. Treatments: E2; 40 µg/kg/day, BPA; 100 mg/kg, OP; 100 mg/kg, ICI; 4 mg/kg.

To determine the effects of BPA and OP on embryo attachment, *MUC1* expression was measured by using both real-time PCR and western-blot assays. As a barrier to implantation, MUC1 expression has an important role in embryo attachment with downregulation of MUC1 expression being necessary for successful implantation [17]. Both *MUC1* mRNA and MUC1 protein levels were markedly higher in the ICI, E2, E2 + ICI, BPA + ICI, OP, and OP + ICI groups (approximate 305%, 2910%, 1321%, 232%, 245%, and 530%, respectively) compared to the VE group (Figure 3a,b). Treatment with BPA did not significantly increase *MUC1* mRNA levels but markedly increased MUC1 protein levels compared to the VE group. The BPA + ICI and OP + ICI combination treatments produced higher MUC1 mRNA expression in uteri compared to the BPA- and OP-alone group, but lower in E2 + ICI groups than in E2 group. These results suggest that OP could impair implantation through the abnormal expression of *MUC1* in uterus. Following implantation success, MUC1 expression was either downregulated or was completely absent [5,16,17,42]. Indeed, the *MUC1* mRNA expression was no change in BPA and OP groups when compared to the VE group in implantation sites (Figure 3c). However, the MUC1 protein level was significantly decreased in BPA group compared to the VE group in implantation sites (Figure 3d). These results suggest that BPA or OP exposure can result in disruption of uterine receptivity. Combined exposure to ICI and BPA or OP can lead to a substantial reduction in embryo attachment.

### 3.4. Effects of BPA and OP on HOXA-10 and LIF Expression in Maternal Uterus and Implantation Sites

**Figure 4:**
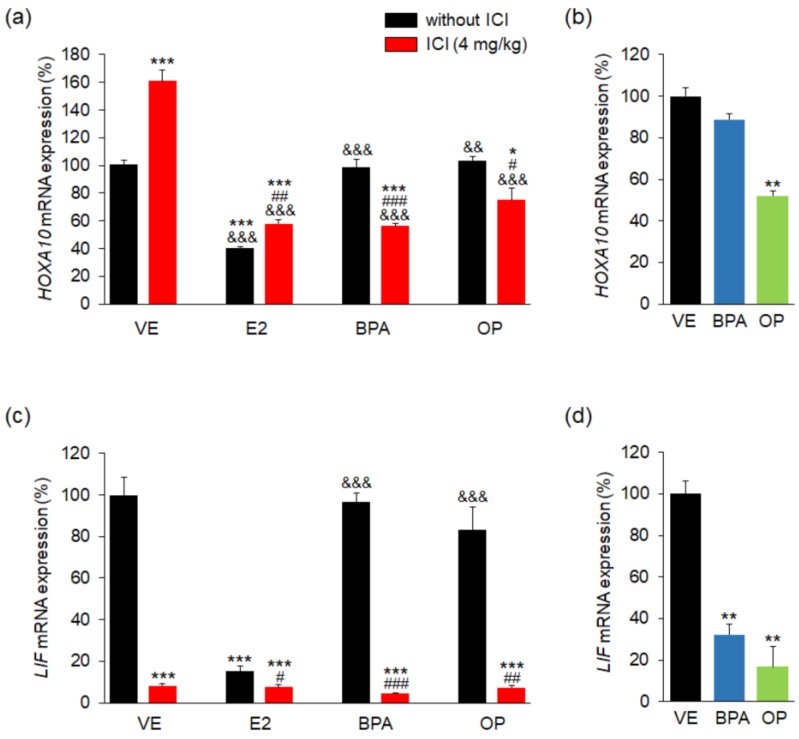
E2, BPA, and OP decreased the expression of *HOXA10* and *LIF* in maternal uterus and implantation sites. The expressions of *HOXA10* and *LIF* genes were measured by real-time PCR and were normalized to that of 18S ribosomal RNA (RN18S). (**a**) mRNA levels of *HOXA10* were significantly different after E2 and EDs + ICI treatment in maternal uterus; (**b**) the mRNA level of *HOXA10* was significantly decreased by OP treatment in implantation sites; (**c**) mRNA levels of *LIF* were significantly low in the ICI, E2 and EDs + ICI groups in maternal uterus (**d**) mRNA levels of *LIF* were markedly lowered by BPA or OP treatment in implantation sites. Both mRNA levels of *HOXA10* and *LIF* were significantly low in the combination BPA + ICI and OP + ICI than that in BPA- and OP-alone in maternal uterus. Statistical significance was determined by two-way ANOVA. * *p* < 0.05 vs. VE, ** *p* < 0.01 vs. VE, *** *p* < 0.001 vs. VE, ^#^ *p* < 0.05 EDs + ICI vs. EDs, ^##^ *p* < 0.01 EDs + ICI vs. EDs, ^###^ *p* < 0.001 EDs + ICI vs. EDs, ^&&^ *p* < 0.01 vs. ICI, ^&&&^ *p* < 0.001 vs. ICI. *n* = 5 mice per group for uterus, *n* = 3 mice per group for implantation sites. Treatments: E2; 40 µg/kg/day, BPA; 100 mg/kg, OP; 100 mg/kg, ICI; 4 mg/kg.

To confirm the effects of BPA and OP on both the maternal uterus and the implantation site, the expressions of the HOXA10 development factor gene and the LIF growth factor gene were measured. The *HOXA10* mRNA level was significantly high in ICI group compared to the VE. In contrast, it was significantly decreased in uteri of the E2, E2 + ICI, BPA + ICI, and OP + ICI groups compared to the uteri of the VE group (Figure 4a). The BPA + ICI and OP + ICI combination treatments reduced lower *HOXA10* mRNA expression in uteri compared to the BPA-, OP-alone group and in ICI group. Moreover, it was markedly lower in the implantation sites of the OP group than in those of the VE group (Figure 4b). There was no significant difference in *HOXA10* expression levels in the implantation sites of the BPA and VE groups.

The expressions of *LIF* mRNA in uterus were markedly lower in the ICI group (92%), E2 + ICI group (93% lower), BPA + ICI and OP + ICI groups (both 95% lower), E2 group (85% lower) compared to the VE group. There was no significant difference in *LIF* mRNA levels in the BPA and OP groups compared to the VE group (Figure 4c). The expression of *LIF* mRNA in uteri was significantly lower in the BPA + ICI and OP + ICI combination treatments compared to the BPA- and OP-alone group. Moreover, *LIF* mRNA levels at implantation sites were significantly lower in the BPA (70% lower) and OP (85% lower) groups compared to the VE group (Figure 4d). These results suggest that both BPA and OP affect development and growth factor expression in both the maternal uterus and implantation sites.

### 3.5. Effects of BPA and OP on Estrogen and Progesterone Receptor in Maternal Uterus and Implantation Sites

**Figure 5:**
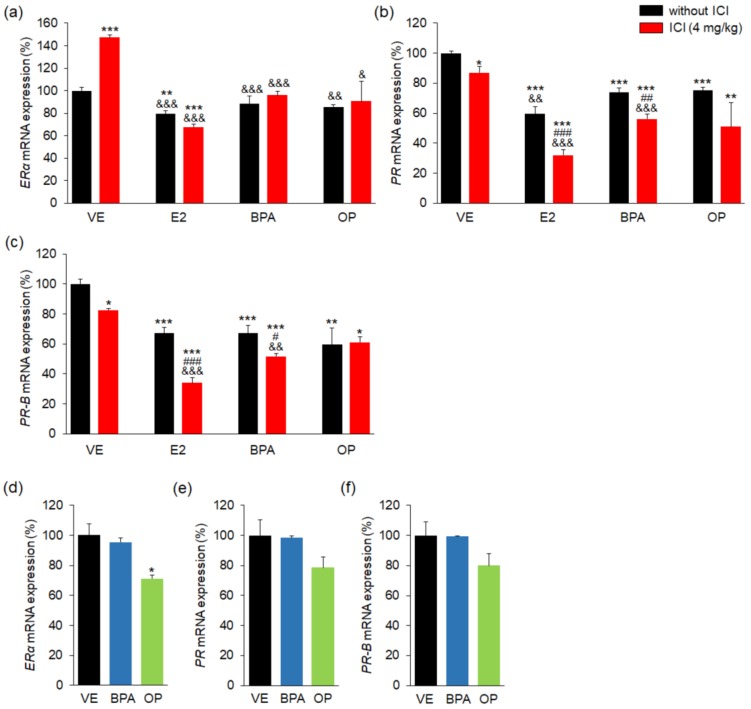
E2, BPA, and OP effects and estrogen and progesterone receptor expressions in maternal uterus and implantation sites. The expression levels of *ERα*, *PRα*, and *PRβ* genes were measured by real-time PCR and normalized to that of 18S ribosomal RNA (RN18S). In uterus, (**a**) mRNA levels of *ER* were not changed by BPA or OP with/without ICI; (**b**) mRNA levels of *PR* were significantly low in all groups; (**c**) mRNA levels of *PR-B* were markedly low in all groups; In implantation sites, (**d**–**f**) the expressions of *ERα* mRNA were significantly decreased by OP. Statistical significance was determined by two-way ANOVA. * *p* < 0.05 vs. VE, ** *p* < 0.01 vs. VE, *** *p* < 0.001 vs. VE, ^#^ *p* < 0.05 EDs + ICI vs. EDs, ^##^ *p* < 0.01 EDs + ICI vs. EDs, ^###^ *p* < 0.001 EDs + ICI vs. EDs, ^&^ *p* < 0.05 vs. ICI, ^&&^ *p* < 0.01 vs. ICI, ^&&&^ *p* < 0.001 vs. ICI. *n* = 5 mice per group for uterus, *n* = 3 mice per group for implantation sites. Treatments: E2; 40 µg/kg/day, BPA; 100 mg/kg, OP; 100 mg/kg, ICI; 4 mg/kg.

Real-time PCR results showed that the expressions of *ERα* mRNA in uterus samples were significantly lower in the E2 and E2 + ICI groups than in the VE group (Figure 5a). Treatment of BPA and OP with/without ICI did not change the expression of *ERα* mRNA compared to VE. The expression of *ERα* mRNA were significantly lower in the E2 and E2 + ICI combination treatments than in the VE group but it was higher in ICI group. Moreover, mRNA level of *PR* in uteri of the ICI, E2, E2 + ICI, BPA, BPA + ICI, OP and OP + ICI groups were markedly lower than that in uteri of the VE group (lower by 13%, 40%, 69%, 36%, 44%, 25% and 42%, respectively) (Figure 5b). The expression of *PR-B* mRNA was also significantly lower in uteri of all groups than in the VE group (Figure 5c). The E2 + ICI, BPA + ICI and OP + ICI combination treatments markedly reduced *PR* and *PR-B* mRNA expression in uteri than that in the E2-, BPA- and ICI-alone group.

At the implantation sites, there were no significant differences in the mRNA levels of *PR* or *PR-B* between the BPA- or OP-treated groups and the VE group (Figure 5d–f). mRNA level of *ERα* of the OP group was significantly lower compared to VE group. These results suggest that BPA and OP can affect blastocyst implantation through modulation of PR in the maternal uterus.

## 4. Discussion

The timing window for blastocyst implantation is a crucial period in the establishment of a successful pregnancy. Abnormal events before, during, or immediately after implantation can result in poor pregnancy rates in many species, including humans. In the present study, we investigated the effect of EDs during the initial pregnancy process. We also investigated the expressions of calcium-related genes and the relationships of their expression to the development of a successful implantation. Indeed, calcium ion is a crucial element in living organisms from fertilization onward. Additionally, the regulation of calcium ion homeostasis clearly plays important roles in the process of implantation. In recent decades, many researchers have reported on the role of the calcium ion during life, particularly during pregnancy. However, the roles of the calcium ion in early pregnancy have not been fully described.

A few studies indicate that exposure to low dose of BPA at early pregnancy did not alter number of litters in mice and rats. Specifically, Xi et al., indicate that gestation day 1 BPA exposure at a dose of 50 mg/kg/day as known as lowest-observed-adverse-effect level (LOAEL) did not affect to litter size in mice [43]. In other words, BPA at LOAEL level did not interfere the development of implantation. Addition, implantation seemed normal in the rest BPA-treated groups or the female offspring from 40 mg/kg/day BPA-treated group [40]. Thus, to clearly understand the pathway that BPA affects to implantation, we used BPA at dose 100 mg/kg that did not showed the difference of the offspring and the number of implantation sites in mice [38,39,40]. But preimplantation 100 mg/kg/day s.c. BPA treatment inhibited embryo implantation and also adversely affected uterine receptivity [40].

By exerting estrogenic activity, EDs can interfere with the normal endocrine process, causing adverse pregnancy outcomes [44]. BPA and OP are among the most disruptive EDs and are widely used as plasticizers in consumer products. Several studies showed that high-dose BPA or OP treatment can result in a lowering of implantation rates in rats and mice [33,45,46]. Moreover, high exposure to BPA has been associated with miscarriage in human [47]. In this study, exposure to EDs resulted in a reduction or loss of implantation success and an abnormal appearance of the implantation site. Notably, direct exposure to ICI has been shown to lead to a complete loss of implantation [41]. Thus, the complete absence of implantation sites in all groups treated with ICI.

The calcium ion is a crucial element in the development and physiology of living organisms and is involved in a variety of important pregnancy-related events, including fertilization, decidualization, and implantation [20]. The calcium ion acts as an intracellular messenger to regulate a diverse range of biological processes related to cellular function, such as gene transcription, proliferation, differentiation, necrosis, and apoptosis [48,49,50]. Thus, maintenance of calcium ion activity in the uterus and at the implantation site is necessary for successful implantation. Calcium ion entry via the calcium channels contributes to calcium ion mobilization, and four cell membrane calcium channel factors, TRPV5, TRPV6, PMCA1, and NCX1 are reported to have critical roles in the major steps of calcium ion transport. TRPV5 and TRPV6 are members of the transient receptor potential cation channel subfamily V gene family and are responsible for the influx of calcium ion into cells during transcellular absorption [51,52]. Plasma membrane Ca^2+^-ATPase 1 (PMCA1) and Na^+^/Ca^2+^ exchanger 1 (NCX1) have important roles in the outflow of cytosolic calcium ions from cells [53,54] and the maintenance of calcium ion level in the cytoplasm [55,56]. Additionally, TRPV5, TRPV6, PMCA1, and NCX1 are not only observed in duodenum and kidney but also in uterus [25,28,57]. Moreover, they have been reported to have important roles in placental calcium ion transport and regulation [58]. These observations suggest that an abnormal expression of a calcium transport gene may induce abnormal maternal–fetal calcium ion transport. For example, exposure to BPA or OP from GD 11.5 to GD 16.5 has induced a decrease in TRPV6 and PMCA1 expression in mouse placenta [35]. Also, BPA and OP can disrupt the expression of calcium transport genes in kidney and duodenum of pregnant mice [34]. Recently, we found that ICI partially down-regulated the TPRV6 gene expression in both mouse and rat uterus during pregnancy [26]. In this study, BPA, OP, and E2 exposure at the initial stage of pregnancy resulted in upregulation of TRPV5 and TRPV6 expressions in uterus. Cotreatment with ICI strictly increased the mRNA expressions of *TRPV5* and *TRPV6*. Interestingly, the expression of *TRPV5*, *TRPV6*, *PMCA1*, and *NCX1* were significantly decreased in the implantation sites of the BPA- and OP-treated groups. These results suggest that exposure to EDs-alone or -combine to ICI during early pregnancy can result in an increase in the intracellular calcium ion level and affect the blastocyst implantation site. Moreover, intracellular calcium ion overload can result in necrotic or apoptotic cell death [59].

The implantation stage is initiated after embryo attachment to the maternal uterus. During attachment, adhesion is the initial step and involves apposition and adhesion of the hatched blastocyst to the uterine luminal epithelium. MUC1 is a transmembrane glycoprotein belonging the mucin family and is expressed at the apical surface of uterine epithelia and various organs. As MUC1 is a barrier to embryo implantation, MUC1 downregulation or absence prior to implantation is a prerequisite for uterine receptivity in many species [5,16,17,42] with downregulation allowing embryo attachment and leading to successful implantation. EDs have been reported to induce the expression of MUC1 and chronic exposure to BPA has resulted in the upregulation of MUC1 during early pregnancy [36]. In the present study, the expression of *MUC1* in uterus was markedly increased by the EDs tested. These results indicate that EDs can affect uterine receptivity and result in failure of blastocyst attachment and implantation. Recently, MUC1 has been shown to increase the expression of TRPV5 in kidney [60]. These results suggest that the expression of TRPV5 and TRPV6 may involve the expression of MUC1 in uterus during pregnancy. Additionally, co-treatment with ICI markedly increased MUC1 gene expression. However, the effect of ICI on the expression of MUC1 is still poor understood.

Implantation success requires the synchronized development of both the embryo and the endometrium. Homeobox gene transcription factors are reported to be crucial for endometrial development and embryo implantation in both mouse and human [10]. *HOXA10*, a member of developmentally regulated AbdB subclass of the homeobox gene, is essential for embryo survival and implantation [61]. Peak *HOXA10* and *HOXA11* expressions are first observed during the window of endometrial receptivity. Decreased *HOXA10* expression leads to a decreased implantation rate; furthermore, *HOXA10* expression is significantly low in cases of recurrent miscarriage and recurrent implantation failure [15]. Recently, it was reported that BPA exposure results in downregulation of *HOXA10* [37]. In this study, the expression of HOXA10 mRNA in uterus was markedly low in E2 and EDs + ICI groups. *HOXA10* mRNA expression in the implantation site was significantly lower in the OP groups. 

Among the cytokines, *LIF* (an interleukin-6 (IL-6) cytokine) is reported to be important for implantation in mouse [4,62]. *LIF* is expressed in both the embryo and the endometrium and is involved in various processes during the implantation period such as blastocyst development, endometrial differentiation, blastocyst attachment, and invasion of the endometrium [4,11]. *LIF* deficiency can significantly decrease the survivability of embryos during the cell and morula stages and can affect blastocyst development [14]. Furthermore, several studies have suggested that *LIF* and *LIF* receptor expressions are significantly low in the endometrium of infertile women [12,13,63]. In this study, *LIF* mRNA levels were significantly lower in the E2, E2 + ICI, BPA + ICI, and OP + ICI groups. Those results support the observed lack of implantation in those groups. Moreover, downregulation of HOXA10 and LIF may be associated with the different appearances of the implantation sites visualized in this study. These results suggest that treatment with EDs can induce the loss of embryo survival via inhibition of the development and growth of the implantation site and through inhibition of uterine preparation.

Both ovarian estrogen (E2) and progesterone (P4) are the principal hormones involved in the preparation of the uterus for embryo implantation and in the maintenance of pregnancy [1,64]. Their synchronized production enables the blastocyst to attach and initiate the implantation process through their effects on uterine structure and function. Embryonic estrogen is considered important for embryo implantation in pigs, guinea pig, rabbits and hamster [1]. E2 and P4 affect the uterus primarily through their nuclear receptors, E2 receptor alpha (*ERα*) and beta (*ERβ*), and via *P4* and *P4* receptor B (*PR-B*), respectively. In mouse, *ERα* is important for uterine receptivity and embryo implantation [65]. In addition, the lack of both *PR* and *PR-B* results in infertility by affecting the function of ovary and uterus. EDs are described as synthetic compounds that mimic natural estrogens, and they can bind to nuclear *ERα*. In addition, *MUC1* and *LIF* expression are regulated by P4 [6], while *HOXA10* and *HOXA11* are upregulated by E2 and P4 [7]. These results suggest that EDs primarily affect embryo implantation, attachment, and survival through the modulation of *PR*, and *PR-B*. 

Throughout experiments, ICI treatments showed the additive effect to BPA and OP. The BPA + ICI and OP + ICI combination strictly increase the expression of TRPV5, TRPV6 and MUC1 genes. In addition, they also markedly decrease the mRNA expression of HOXA10, LIF, PR and PR-B compared to the BPA- and OP-alone. However, the effects of ICI-exposure is not fully understood; ICI can act both as an agonist and antagonist of estradiol effects [66,67,68]. Therefore, we will focus on assessing ICI-exposure alone in future studies.

## 5. Conclusions

These results show that BPA and OP regulate the expression of TRPV5, TRPV6, PMCA1, and NCX1 in both maternal uterus and embryo implantation sites during the implantation stage in mouse. Additionally, they disrupt orchestration of embryo–uterine cross-talk by modulating the expression of *MUC1*, *LIF*, and *HOXA10* genes. Moreover, there are several reports showing that *LIF* and *MUC1* can regulate the calcium transient transport channel [18,19]. Thus, overexpressions of TRPV5 and TRPV6 appear to be involved in the downregulation of *LIF* and upregulation of *MUC1* in uterus. As a consequence, BPA and OP can reduce the implantation rate. In conclusion, BPA, OP, and E2 can have negative effects on the embryo implantation and survival by disrupting the calcium transient transport channel and by affecting growth and development factors through the progesterone receptor.

## Supplementary Materials

The following are available online at http://www.mdpi.com/1660-4601/15/8/1614/s1, Figure S1: Pregnant mice at gestation day (GD) 5.5 were sacrificed 48 h after injection. Implantation sites in uteri were detected by administration of Chicago Sky Blue 5 min before sacrifice. All implantation sites in the control group were detected as distinct blue bands. Images are representative of implanted blastocysts in uteri. Scare bar = 1 cm. The implantation sites in the uterus are indicated by the arrow. N = 3 mice per group, Figure S2: Mice were sacrificed at GD 4.5 (24 h after final injection) to collect uterus tissues and at GD 5.5 (48 h after final injection) to collect implantation sites. Protein expression of calcium transporter channel genes in uterus and implantation sites were assessed. Expressions of TRPV5, TRPV6, PMCA1, and NCX1 proteins were investigated by western blotting and results were normalized to β-actin. Histograms show quantification of blots. In uterus, (a,b) protein levels of TRPV5 and TRPV6 were markedly high in all groups. (c) There were no changes in PMCA1 expression. (d) Protein levels of NCX1 were significantly increased by E2. In implantation sites, (e and f) TRPV5 and TRPV6 protein levels were significantly decreased by OP treatment. (g,h) PMCA1 and NCX1 protein levels were decreased but not significantly. Statistical significance was determined by two-way ANOVA. * *p* < 0.05 vs. VE, ** *p* < 0.01 vs. VE, *** *p* < 0.001 vs. VE, ^###^ *p* < 0.001 EDs + ICI vs. EDs, ^&&^ *p* < 0.01 vs. ICI. *n* = 5 mice per group for uterus, *n* = 3 mice per group for implantation sites. Treatments: E2; 40 µg/kg/day, BPA; 100 mg/kg, OP; 100 mg/kg, ICI; 4 mg/kg.

## Abbreviations

Bisphenol A	(BPA; 4,4′-(propane-2,2-diyl)diphenol)
OP	4-*tert*-Octylphenol
E2	17β-Estradiol
P4	Progesterone
ICI	ICI 182,780
EDs	Endocrine disrupting chemicals
TRPV5 and TRPV6	Transient receptor potential cation channel subfamily V (TRPV) member 5 and 6
PMCA1	Plasma membrane Ca^2+^-ATPase 1
NCX1	Na^+^/Ca^2+^ exchanger 1
LIF	Leukemia inhibitory factor
HOXA-10	homebox gene member 10
MUC1	Mucin 1
ERα and ERβ	Estrogen receptor alpha and beta
PR- and PR-B	Progesterone receptor and progesterone receptor B
LOAEL	Lowest-observed-adverse-effect level

## References

[1] Dey S., Lim H., Das S.K., Reese J., Paria B., Daikoku T., Wang H. (2004). Molecular cues to implantation. Endocr. Rev..

[2] Wang H., Dey S.K. (2006). Roadmap to embryo implantation: Clues from mouse models. Nat. Rev. Genet..

[3] Norwitz E.R., Schust D.J., Fisher S.J. (2001). Implantation and the survival of early pregnancy. N. Engl. J. Med..

[4] Paiva P., Menkhorst E., Salamonsen L., Dimitriadis E. (2009). Leukemia inhibitory factor and interleukin-11: Critical regulators in the establishment of pregnancy. Cytokine Growth Factor Rev..

[5] Surveyor G.A., Gendler S.J., Pemberton L., Das S., Chakraborty I., Julian J., Pimental R., Wegner C., Dey S., Carson D. (1995). Expression and steroid hormonal control of Muc-1 in the mouse uterus. Endocrinology.

[6] Meseguer M., Aplin J.D., Caballero-Campo P., O’Connor J.E., Martín J.C., Remohí J., Pellicer A., Simón C. (2001). Human endometrial mucin MUC1 is up-regulated by progesterone and down-regulated in vitro by the human blastocyst. Biol. Reprod..

[7] Taylor H.S., Arici A., Olive D., Igarashi P. (1998). HOXA10 is expressed in response to sex steroids at the time of implantation in the human endometrium. J. Clin. Investig..

[8] Lee J.Y., Lee M., Lee S.K. (2011). Role of endometrial immune cells in implantation. Clin. Exp. Reprod. Med..

[9] Ren L., Marquardt M.A., Lech J.J. (1997). Estrogenic effects of nonylphenol on pS2, ER and MUC1 gene expression in human breast cancer cells-MCF-7. Chem.-Biol. Interact..

[10] Du H., Taylor H.S. (2016). The role of Hox genes in female reproductive tract development, adult function, and fertility. Cold Spring Harbor Perspect. Med..

[11] Aghajanova L. (2004). Leukemia inhibitory factor and human embryo implantation. Ann. N. Y. Acad. Sci..

[12] Hambartsoumian E. (1998). Endometrial leukemia inhibitory factor (LIF) as a possible cause of unexplained infertility and multiple failures of implantation. Am. J. Reprod. Immunol..

[13] Alizadeh Z., Shokrzadeh N., Saidijam M., Sanoee M.F. (2011). Semi-quantitative analysis of HOXA11, leukemia inhibitory factor and basic transcriptional element binding protein 1 mRNA expression in the mid-secretory endometrium of patients with endometriosis. Iran. Biomed. J..

[14] Cheng T.-C., Huang C.-C., Chen C.-I., Liu C.-H., Hsieh Y.-S., Huang C.-Y., Lee M.-S., Liu J.-Y. (2004). Leukemia inhibitory factor antisense oligonucleotide inhibits the development of murine embryos at preimplantation stages. Biol. Reprod..

[15] Yang Y., Chen X., Saravelos S.H., Liu Y., Huang J., Zhang J., Li T.C. (2017). HOXA-10 and E-cadherin expression in the endometrium of women with recurrent implantation failure and recurrent miscarriage. Fertil. Steril..

[16] DeSouza M.M., Mani S.K., Julian J., Carson D.D. (1998). Reduction of mucin-1 expression during the receptive phase in the rat uterus. Biol. Reprod..

[17] Bowen J.A., Bazer F.W., Burghardt R.C. (1996). Spatial and temporal analyses of integrin and Muc-1 expression in porcine uterine epithelium and trophectoderm in vivo. Biol. Reprod..

[18] Murata M., Fukuda K., Ishida H., Miyoshi S., Koura T., Kodama H., Nakazawa H.K., Ogawa S. (1999). Leukemia inhibitory factor, a potent cardiac hypertrophic cytokine, enhances L-type Ca^2+^ current and [Ca^2+^] i transient in cardiomyocytes. J. Mol. Cell. Cardiol..

[19] Guang W., Kim K.C., Lillehoj E.P. (2009). MUC1 mucin interacts with calcium-modulating cyclophilin ligand. Int. J. Biochem. Cell Biol..

[20] Banerjee A., Padh H., Nivsarkar M. (2009). Role of the calcium channel in blastocyst implantation: A novel contraceptive target. J. Basic Clin. Physiol. Pharmacol..

[21] Whitaker M. (2006). Calcium at fertilization and in early development. Physiol. Rev..

[22] Santella L., Lim D., Moccia F. (2004). Calcium and fertilization: The beginning of life. Trends Biochem. Sci..

[23] Peng J.-B., Brown E.M., Hediger M.A. (2003). Apical entry channels in calcium-transporting epithelia. Physiology.

[24] Van Abel M., Hoenderop J.G., Bindels R.J. (2005). The epithelial calcium channels *TRPV5* and *TRPV6*: Regulation and implications for disease. Naunyn-Schmiedeberg’s Arch. Pharmacol..

[25] Kim J.-A., Yang H., Hwang I., Jung E.-M., Choi K.-C., Jeung E.-B. (2011). Expression patterns and potential action of the calcium transport genes *TRPV5, TRPV6*, *NCX1* and *PMCA1B* in the canine duodenum, kidney and uterus. In Vivo.

[26] Lee B.-M., Lee G.-S., Jung E.-M., Choi K.-C., Jeung E.-B. (2009). Uterine and placental expression of TRPV6 gene is regulated via progesterone receptor-or estrogen receptor-mediated pathways during pregnancy in rodents. Reprod. Biol. Endocrinol..

[27] Sprekeler N., Kowalewski M.P., Boos A. (2012). TRPV6 and Calbindin-D9k-expression and localization in the bovine uterus and placenta during pregnancy. Reprod. Biol. Endocrinol..

[28] Yang H., Choi K.C., Hyun S.H., Jeung E.B. (2011). Coexpression and estrogen-mediated regulation of *TRPV6* and PMCA1 in the human endometrium during the menstrual cycle. Mol. Reprod. Dev..

[29] Gore A.C., Chappell V., Fenton S., Flaws J.A., Nadal A., Prins G.S., Toppari J., Zoeller R. (2015). EDC-2: The endocrine society’s second scientific statement on endocrine-disrupting chemicals. Endocr. Rev..

[30] Woodruff T.J., Carlson A., Schwartz J.M., Giudice L.C. (2008). Proceedings of the summit on environmental challenges to reproductive health and fertility: Executive summary. Fertil. Steril..

[31] Yuan M., Bai M.-Z., Huang X.-F., Zhang Y., Liu J., Hu M.-H., Zheng W.-Q., Jin F. (2015). Preimplantation exposure to bisphenol A and triclosan may lead to implantation failure in humans. BioMed Res. Int..

[32] Berger R.G., Shaw J. (2008). Impact of acute bisphenol-A exposure upon intrauterine implantation of fertilized ova and urinary levels of progesterone and 17β-estradiol. Reprod. Toxicol..

[33] Harazono A., Ema M. (2001). Effects of 4-tert-octylphenol on initiation and maintenance of pregnancy following oral administration during early pregnancy in rats. Toxicol. Lett..

[34] Kim S., An B.-S., Yang H., Jeung E.-B. (2013). Effects of octylphenol and bisphenol A on the expression of calcium transport genes in the mouse duodenum and kidney during pregnancy. Toxicology.

[35] Lee J.-H., Ahn C., Kang H.Y., Hong E.-J., Hyun S.-H., Choi K.-C., Jeung E.-B. (2016). Effects of octylphenol and bisphenol A on the metal cation transporter channels of mouse placentas. Int. J. Environ. Res. Public Health.

[36] Li Q., Davila J., Bagchi M.K., Bagchi I.C. (2016). Chronic exposure to bisphenol a impairs progesterone receptor-mediated signaling in the uterus during early pregnancy. Recept. Clin. Investig..

[37] Varayoud J., Ramos J.G., Bosquiazzo V.L., Lower M., Muñoz-de-Toro M., Luque E.H. (2011). Neonatal exposure to bisphenol A alters rat uterine implantation-associated gene expression and reduces the number of implantation sites. Endocrinology.

[38] Suzuki A., Sugihara A., Uchida K., Sato T., Ohta Y., Katsu Y., Watanabe H., Iguchi T. (2002). Developmental effects of perinatal exposure to bisphenol-A and diethylstilbestrol on reproductive organs in female mice. Reprod. Toxicol..

[39] Berger R.G., Foster W.G. (2010). Bisphenol-A exposure during the period of blastocyst implantation alters uterine morphology and perturbs measures of estrogen and progesterone receptor expression in mice. Reprod. Toxicol..

[40] Xiao S., Diao H., Smith M.A., Song X., Ye X. (2011). Preimplantation exposure to bisphenol A (BPA) affects embryo transport, preimplantation embryo development, and uterine receptivity in mice. Reprod. Toxicol..

[41] Kumar R., Yadav A., Pakrasi P. (2017). Expression of ER-α and ER-β during peri-implantation period in uterus is essential for implantation and decidualization in golden hamster. Life Sci..

[42] Hild-Petito S., Fazleabas A.T., Julian J., Carson D.D. (1996). Mucin (Muc-1) expression is differentially regulated in uterine luminal and glandular epithelia of the baboon (Papio anubis). Biol. Reprod..

[43] Xi W., Lee C., Yeung W., Giesy J.P., Wong M., Zhang X., Hecker M., Wong C.K. (2011). Effect of perinatal and postnatal bisphenol A exposure to the regulatory circuits at the hypothalamus–pituitary–gonadal axis of CD-1 mice. Reprod. Toxicol..

[44] Bengtsson J., Thygesen P.S., Kaerlev L., Knudsen L.E., Bonde J.P. (2017). Potential exposure to endocrine disrupting chemicals and selected adverse pregnancy outcomes: A follow-up study of pregnant women referred for occupational counselling. J. Occup. Med. Toxicol..

[45] Berger R.G., Hancock T. (2007). Influence of oral and subcutaneous bisphenol-A on intrauterine implantation of fertilized ova in inseminated female mice. Reprod. Toxicol..

[46] Borman E.D., Foster W.G., Greenacre M.K., Muir C.C. (2015). Stress lowers the threshold dose at which bisphenol A disrupts blastocyst implantation, in conjunction with decreased uterine closure and e-cadherin. Chem.-Biol. Interact..

[47] Sugiura-Ogasawara M., Ozaki Y., Sonta S.-I., Makino T., Suzumori K. (2005). Exposure to bisphenol A is associated with recurrent miscarriage. Hum. Reprod..

[48] Berridge M.J., Lipp P., Bootman M.D. (2000). The versatility and universality of calcium signalling. Nat. Rev. Mol. Cell Biol..

[49] Berridge M.J., Bootman M.D., Roderick H.L. (2003). Calcium signalling: Dynamics, homeostasis and remodelling. Nat. Rev. Mol. Cell Biol..

[50] Clapham D.E. (2007). Calcium signaling. Cell.

[51] Hoenderop J.G., van der Kemp A.W., Hartog A., van de Graaf S.F., van Os C.H., Willems P.H., Bindels R.J. (1999). Molecular identification of the apical Ca^2+^ channel in 1, 25-dihydroxyvitamin D3-responsive epithelia. J. Biol. Chem..

[52] Peng J.-B., Chen X.-Z., Berger U.V., Vassilev P.M., Tsukaguchi H., Brown E.M., Hediger M.A. (1999). Molecular cloning and characterization of a channel-like transporter mediating intestinal calcium absorption. J. Biol. Chem..

[53] Zoccola D., Tambutté E., Kulhanek E., Puverel S., Scimeca J.-C., Allemand D., Tambutté S. (2004). Molecular cloning and localization of a PMCA P-type calcium ATPase from the coral Stylophora pistillata. Biochim. Biophys. Acta.

[54] Linck B., Qiu Z., He Z., Tong Q., Hilgemann D.W., Philipson K.D. (1998). Functional comparison of the three isoforms of the Na^+^/Ca^2+^ exchanger (NCX1, NCX2, NCX3). Am. J. Physiol.-Cell Physiol..

[55] Żylińska L., Kawecka I., Lachowicz L., Szemraj J. (2002). The isoform-and location-dependence of the functioning of the plasma membrane calcium pump. Cell. Mol. Biol. Lett..

[56] Blaustein M.P. (2009). Sodium/calcium exchange. Handbook of Cell Signaling.

[57] Choi K., An B., Yang H., Jeung E. (2011). Regulation and molecular mechanisms of calcium transport genes: Do they play a role in calcium transport in the uterine endometrium. J. Physiol. Pharmacol..

[58] Belkacemi L., Bédard I., Simoneau L., Lafond J. (2005). Calcium channels, transporters and exchangers in placenta: A review. Cell Calcium.

[59] Orrenius S., Nicotera P. (1994). The calcium ion and cell death. J. Neural Transm. Suppl..

[60] Nie M., Bal M.S., Yang Z., Liu J., Rivera C., Wenzel A., Beck B.B., Sakhaee K., Marciano D.K., Wolf M.T. (2016). Mucin-1 increases renal TRPV5 activity in vitro, and urinary level associates with calcium nephrolithiasis in patients. J. Am. Soc. Nephrol..

[61] Satokata I., Benson G., Maas R. (1995). Sexually dimorphic sterility phenotypes in HoxalO-deficient mice. Nature.

[62] Stewart C.L., Kaspar P., Brunet L.J., Bhatt H., Gadi I., Köntgen F., Abbondanzo S.J. (1992). Blastocyst implantation depends on maternal expression of leukaemia inhibitory factor. Nature.

[63] Margioula-Siarkou C., Prapas Y., Petousis S., Milias S., Ravanos K., Kalogiannidis I., Mavromatidis G., Haitoglou C., Prapas N., Rousso D. (2016). LIF and LIF-R expression in the endometrium of fertile and infertile women: A prospective observational case-control study. Mol. Med. Rep..

[64] Cha J., Sun X., Dey S.K. (2012). Mechanisms of implantation: Strategies for successful pregnancy. Nat. Med..

[65] Lubahn D.B., Moyer J.S., Golding T.S., Couse J.F., Korach K.S., Smithies O. (1993). Alteration of reproductive function but not prenatal sexual development after insertional disruption of the mouse estrogen receptor gene. Proc. Natl. Acad. Sci. USA.

[66] Robertson J.A., Zhang Y., Ing N.H. (2001). ICI 182,780 acts as a partial agonist and antagonist of estradiol effects in specific cells of the sheep uterus. J. Steroid Biochem. Mol. Biol..

[67] Movérare-Skrtic S., Börjesson A.E., Farman H.H., Sjögren K., Windahl S.H., Lagerquist M.K., Andersson A., Stubelius A., Carlsten H., Gustafsson J.-Å. (2014). The estrogen receptor antagonist ICI 182,780 can act both as an agonist and an inverse agonist when estrogen receptor α AF-2 is modified. Proc. Natl. Acad. Sci. USA.

[68] Chen Y., Li Z., He Y., Shang D., Pan J., Wang H., Chen H., Zhu Z., Wan L., Wang X. (2014). Estrogen and pure antiestrogen fulvestrant (ICI 182 780) augment cell–matrigel adhesion of MCF-7 breast cancer cells through a novel G protein coupled estrogen receptor (GPR30)-to-calpain signaling axis. Toxicol. Appl. Pharmacol..

