# Supplementary material for: Effects of Bisphenol A and 4-tert-Octylphenol on Embryo Implantation Failure in Mouse
Source: Int J Environ Res Public Health. 2018 Jul 30;15(8):1614. doi: 10.3390/ijerph15081614 (PMC6121468; doi:10.3390/ijerph15081614)
Supplement: Supplementary file 1 [file ijerph-15-01614-s001.pdf]

International Journal of
Environmental Research and Public Health

MDPI

Supplementary Material

Effects of Bisphenol A and 4-*tert*-Octylphenol on Embryo Implantation Failure in Mouse

Dinh Nam Tran, Eui-Man Jung, Changhwan Ahn, Jae-Hwan Lee, Yeong-Min Yoo and Eui-Bae Jeung *

Laboratory of Veterinary Biochemistry and Molecular Biology, Veterinary Medical Center and College of Veterinary Medicine, Chungbuk National University, Cheongju 28644, Chungbuk, Korea; mr.tran90tb@gmail.com (D.N.T.); jemman@hanmail.net (E.-M.J.); prac@naver.com (C.A.); phantom4015@nate.com (J.-H.L.); yyeongm@hanmail.net (Y.-M.Y.)

* Correspondence: ebjeung@chungbuk.ac.kr; Tel.: +82-43-261-2397; Fax: +82-43-267-3150

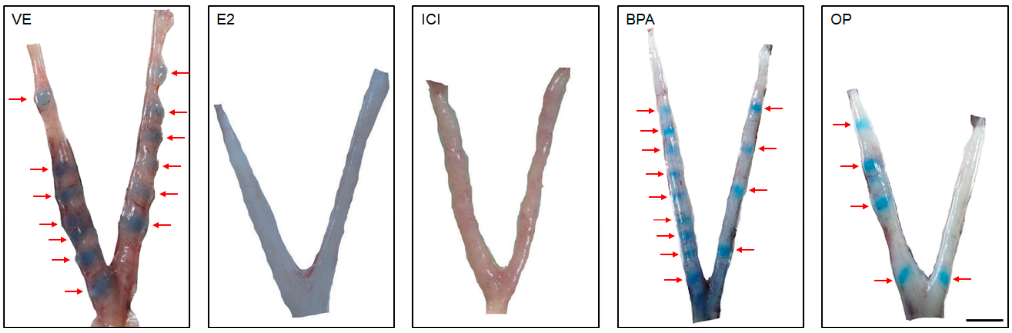

Supplementary Figure S1. Pregnant mice at gestation day (GD) 5.5 were sacrificed 48 h after injection. Implantation sites in uteri were detected by administration of Chicago Sky Blue 5 min before sacrifice. All implantation sites in the control group were detected as distinct blue bands. Images are representative of implanted blastocysts in uteri. Scare bar = 1cm. The implantation sites in the uterus are indicated by the arrow. n = 3 mice per group.

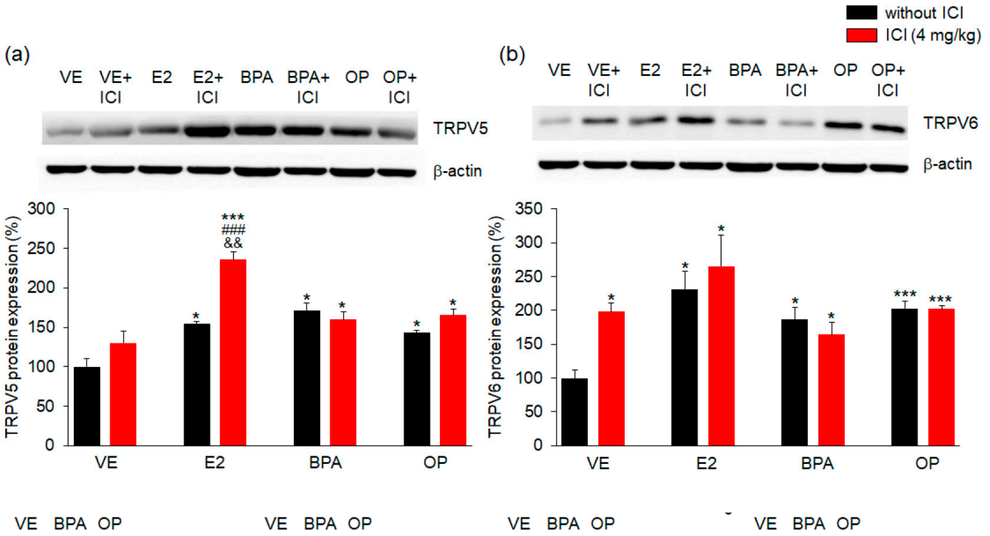

Figure S2. *Cont.*

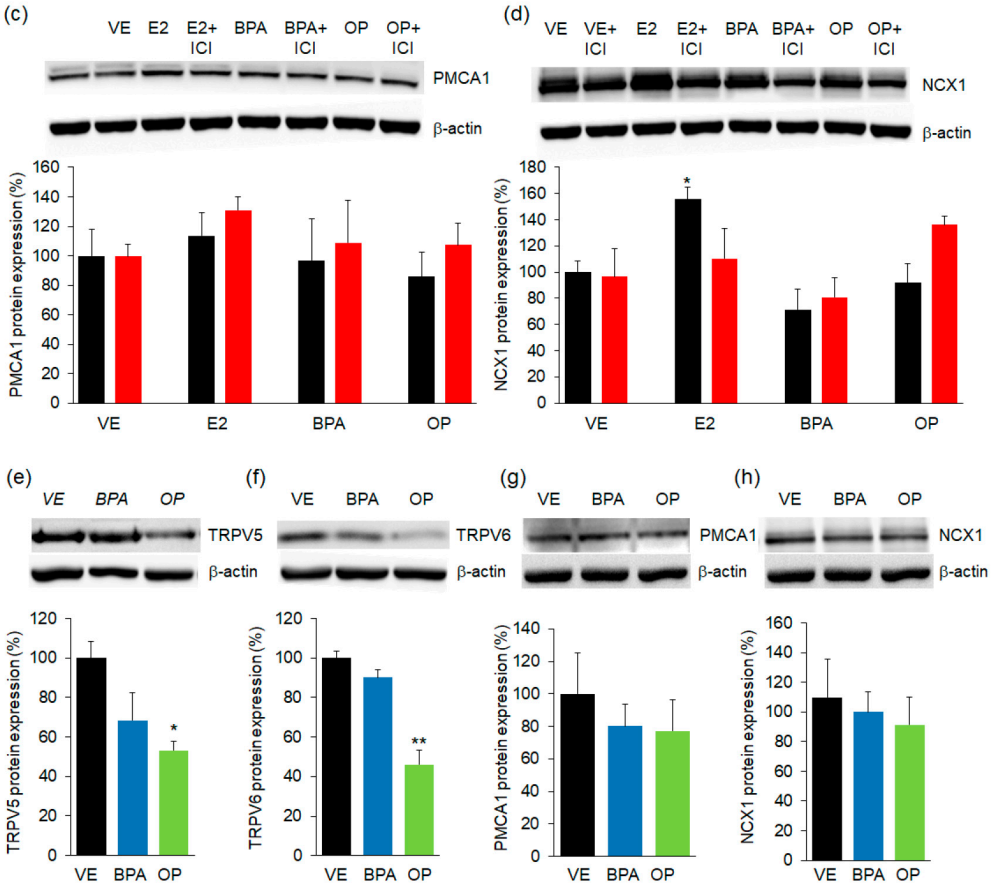

Supplementary Figure S2. Mice were sacrificed at GD 4.5 (24 h after final injection) to collect uterus tissues and at GD 5.5 (48 h after final injection) to collect implantation sites. Protein expression of calcium transporter channel genes in uterus and implantation sites were assessed. Expressions of TRPV5, TRPV6, PMCA1, and NCX1 proteins were investigated by western blotting and results were normalized to β-actin. Histograms show quantification of blots. In uterus, (**a** and **b**) protein levels of TRPV5 and TRPV6 were markedly high in all groups. (**c**) There were no changes in PMCA1 expression. (**d**) Protein levels of NCX1 were significantly increased by E2. In implantation sites, (**e** and **f**) TRPV5 and TRPV6 protein levels were significantly decreased by OP treatment. (**g** and **h**) PMCA1 and NCX1 protein levels were decreased but not significantly. Statistical significance was determined by two-way ANOVA. $^{*}p < 0.05$ vs. VE, $^{**}p < 0.01$ vs. VE, $^{***}p < 0.001$ vs. VE, $^{\#\#\#}p < 0.001$ EDs+ICI vs. EDs, $^{\&\&}p < 0.01$ vs. ICI. $n = 5$ mice per group for uterus, $n = 3$ mice per group for implantation sites. Treatments: E2; 40 μg/kg/day, BPA; 100 mg/kg, OP; 100 mg/kg, ICI; 4 mg/kg.

Supplementary Table. Primer sequences for real-time PCR

Gene	Primer sequence (5`→ 3`)	Accession No.
TRPV5	F: CGTACACATGTTCGAGATAACACC	NM_001007572.2
	R: AGATGTCTGGGATCTCTAGGAGT	
TRPV6	F: GCTGGTTCTTGAGGGTGGAA	NM_022413.4
	R: ATAGGCACCAAAGGGACGTG	
PMCA1	F: GCACCAAGTTGAAAACATCTCCC	NM_026482.2
	R: TCTCCACAAAGTGCATTATCCCC	
NCX1	F: TCAGGAAGGTCCATGCTAGAGA	NM_001112798.2
	R: CCACAGTGCTCTTGAATTCGT	
MUC1	F: GGTTGCTTTGGCTATCGTCT	NM_013605.2
	R: ATTACCTGCCGAAACCTCCT	
HOXA10	F: TGAGTCAAGGCAGTTCCAAAG	NM_001122950.2
	R: TTCACTTGTCTGTCCGTGAGG	
LIF	F: AGGGATTGTGCCCTTACTGC	NM_008501.2
	R: TCCCCTTGAGCTGTGTAATAGG	
ERα	F: TGTGTCCAGCTACAAACCAATG	NM_001302531.1
	R: CATCATGCCCACTTCGTAACA	
PR	F: AATCCCACAGGAGTTTGTCA	NM_008829.2
	R: GGACAACCCCTTTCTGTCTT	
PR-B	F: GGTCCCCCTTGCTTGCA	NM_008829.2
	R: CAGGACCGAGGAAAAAGCAG	
18s rRNA	F: AGACTGTGTCCCTGTGGAGA	NR_046237.1
	R: GGACACGGACAGGATTGACA	
